# Recent developments and trends in the iron- and cobalt-catalyzed Sonogashira reactions

**DOI:** 10.3762/bjoc.18.31

**Published:** 2022-03-03

**Authors:** Surendran Amrutha, Sankaran Radhika, Gopinathan Anilkumar

**Affiliations:** 1 Institute for Integrated Programmes and Research in Basic Sciences (IIRBS), Mahatma Gandhi University, Priyadarsini Hills P O, Kottayam, Kerala, 686560, India; 2 School of Chemical Sciences, Mahatma Gandhi University, Priyadarsini Hills P O, Kottayam, Kerala, 686560, India

**Keywords:** C–C bond formation, cobalt, green reaction, iron, nanoparticles, Sonogashira

## Abstract

Iron- and cobalt-catalyzed Sonogashira coupling reactions are becoming central areas of research in organic synthesis. Owing to their significant importance in the formation of carbon–carbon bonds, numerous green and nanoparticle protocols have emerged during the past decades. The non-toxic and inexpensive nature of catalysts gained much attention in recent times. In this context, their catalytic nature and activity in Sonogashira coupling reactions were well explored and compared. Most importantly, one of the highlights of this review is the emphasis given to green strategies. This is the first review on iron- and cobalt-catalyzed Sonogashira coupling reactions which comprehends literature up to 2020.

## Introduction

The palladium-catalyzed cross-coupling reaction between an aryl/vinyl halide (Cl, Br, I, OTf) and a terminal alkyne in the presence of a Cu(I) co-catalyst under basic conditions to form a Csp^2^–Csp bond generating an arylalkyne is known as the Sonogashira (Sonogashira–Hagihara) coupling [1] and has become an important C–C bond-forming reaction [2]. Over the past 15 years, there has been a growing interest in the Sonogashira coupling reaction, which is one of the most powerful methods for the formation of Csp^2^–Csp bonds leading to arylalkynes and conjugated alkenynes, which are often intermediates or precursors in the synthesis of natural products, pharmaceuticals of biological activity, and materials science [3]. In a Sonogashira reaction, the reaction conditions are mild and many reactions can be performed at room temperature [4]. However, the reaction is usually carried out in organic solvents like amines, benzene, THF, and DMF along with palladium catalysts, that are expensive and sometimes difficult to manipulate and recover [5]. Due to the importance of arylalkynes and alkenynes, the development of environmentally benign, economical, practical, and efficient catalytic systems has received considerable attention.

Back in 2008, Chinchilla et al. reviewed the Sonogashira reaction demonstrating its wide generality and applicability, and the protocol has become a booming methodology in synthetic organic chemistry [6]. Thereafter, the same authors again compiled the Sonogashira reaction covering literature up to 2011 attesting the importance of this coupling reaction [7].

Later, Sonogashira-type reactions requiring only copper as catalyst alone [8] and with other transition metals [9–12] have been reported. Especially iron has attracted a great deal of attention owing to its low price, easy availability, abundant nature, and exceptional versatility [13–15]. Therefore, the low cost first series transition metals such as iron and cobalt show higher significance than other transition metals. Naturally, Sonogashira cross-coupling reactions using cobalt or iron catalysts were reported as more cost-effective alternatives to the original Sonogashira coupling. Both, iron and cobalt catalysts are considered environmentally friendly as they are non-toxic and inexpensive, and thus have significance in modern organic synthesis. The use of an aqueous medium in Fe-catalyzed reactions represents the most reasonable and green option for organic transformations [16]. The development of improved procedures in which less expensive and more suitable catalysts are used has remained an elusive goal. In this respect, iron catalysts stand out as valuable alternatives to those transition metals used in Sonogashira coupling reactions [17].

With growing apprehensions regarding the costs and environment, replacing non-green precious metal catalysts with most advanced green metals is very desirable and attractive [18].

The exploration of non-noble metals like iron, provides a green outlook in catalysis due to its relative abundance and eco-friendly nature. Furthermore, the 3d transition metals show contrast in the reactive nature with 4d and 5d group members [19]. Catalysts based on Earth abundant metals such as Fe and Co are promising members of the 3d series in catalysis [20]. Recently, catalysts based on iron and cobalt complexes have been applied in Sonogashira coupling reactions run in green solvents. The substantial increment of prices for many transition and rare earth metals over the past decade demands more affordable alternatives. This study thus opens the path towards the development of green protocols and application of green catalytic system was still in high need.

To the best of our knowledge, no review has yet been reported on Sonogashira-type reactions catalyzed by Fe or Co catalysts. In this context, we have compiled this first review on iron- and cobalt-catalyzed Sonogashira coupling reactions comprehending the advances, trends, and perspectives in this field, covering literature up to 2020.

## Review

### Classification

For simplicity and easy understanding, the review is divided mainly into two sections as 1) iron-catalyzed Sonogashira coupling reactions and 2) cobalt-catalyzed Sonogashira coupling reactions, with each section further classified. The Fe-catalyzed Sonogashira coupling reactions are subdivided into green protocols and miscellaneous protocols while the Co-catalyzed Sonogashira coupling reactions are further divided into nanoparticle-based strategies and non-nanoparticle-based methods.

### Fe-catalyzed Sonogashira cross-coupling reactions

#### Homogeneous green protocols

**Scheme 1:**
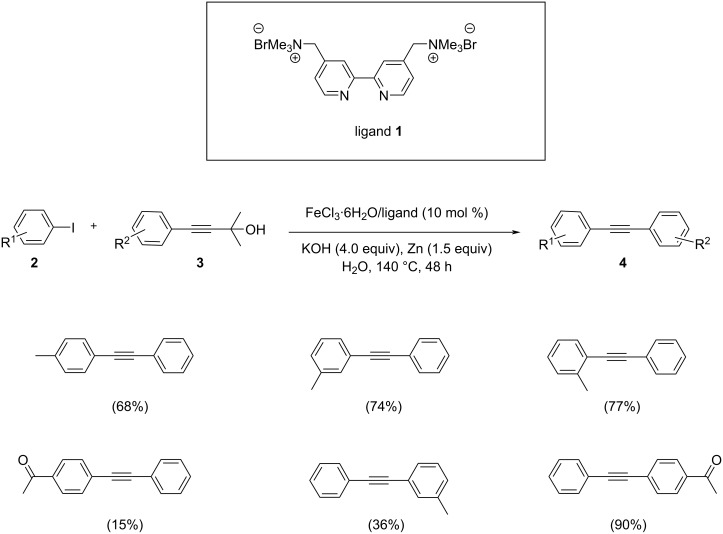
One pot Sonogashira coupling of aryl iodides with arylynols in the presence of iron(III) chloride hexahydrate.

Tsai et al. discussed an efficient, simple and environmentally friendly method for the coupling of arylynols **3** with an aryl halide [21]. This strategy discloses a one pot reaction catalyzed by FeCl_3_ in an aqueous medium associated with the cationic ligand 2,2’-bipyridyl (Scheme 1). The reaction utilizes KOH as the base, since it provides the suitable deprotection of arylynols **3** during the synthesis of the terminal arylalkynes. The optimized conditions revealed that 10 mol % of Fe catalyst/ligand at 140 °C for 48 h afforded the maximum yields of the products. Both, asymmetric and symmetric alkynes were synthesized by this one pot method without the use of any organic solvent. For a better yield of the coupled alkyne product, Zn powder was used as a reductant. Lower yields were obtained by the use of *ortho*-substituted arylynols as the substrate due to steric effects.

**Scheme 2:**
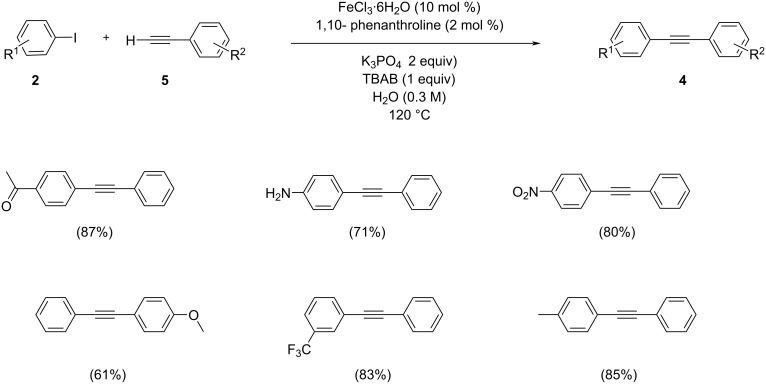
The iron-catalyzed Sonogashira coupling of aryl iodides with terminal acetylenes in water under aerobic conditions.

Anilkumar and co-workers reported an iron-catalyzed Sonogashira coupling of aryl iodides with terminal alkynes in the presence of a catalytic system made up of the greenest solvent, water, in the presence of 10 mol % FeCl_3_·6H_2_O and 20 mol % 1,10-phenanthroline as ligand under aerobic conditions [13]. This system is applicable to sterically hindered aryl iodides or substituted aryl iodides with terminal arylalkynes (Scheme 2). K_3_PO_4_ was the effective base for the reaction of 4-iodoacetophenone with phenylacetylene. Ligands such as DABCO and *trans*-1,2-diaminocyclohexane were also tested, but their efficiencies were found to be lower than that of 1,10-phenanthroline. Metal impurities in FeCl_3_·6H_2_O were detected by using ICP mass spectrometry.

**Scheme 3:**
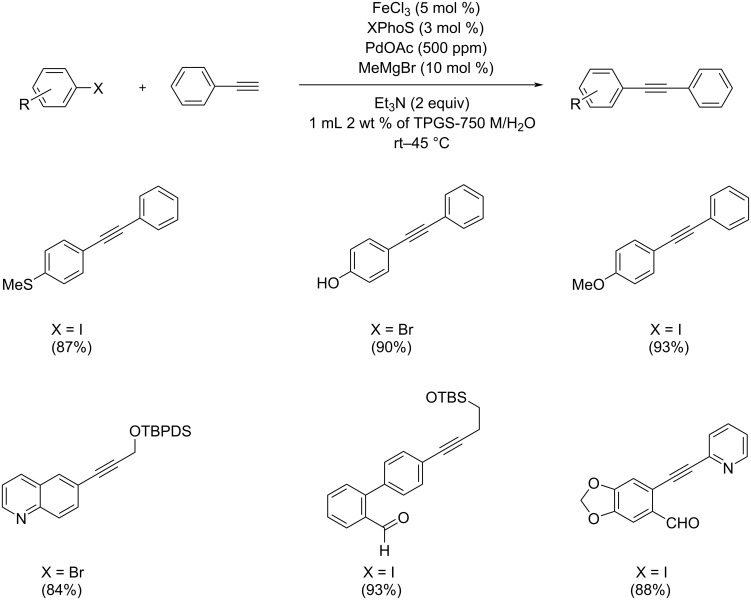
Sonogashira coupling of aryl halides and phenylacetylene in the presence of iron nanoparticles.

Lipshutz and co-workers prepared nanoparticles from inexpensive iron(III) chloride containing reusable palladium in ppm level and XPhoS as ligand for the effective Sonogashira coupling under aqueous conditions (Scheme 3) [22]. The coupling reaction was catalyzed by the nanoparticles in water between room temperature and 45 °C. Notably, the amount of Pd was below the detection limit. The reaction done in the presence of 1000 ppm of Pd ligated with XPhoS provided only 12% of the product. Both, Fe and Pd are essentially required for the successive coupling in nanocomposites, and hence iron composition plays a major role in the activity of the nanoparticles. Selective reaction between an aryl iodide/bromide with the terminal alkyne was facile in the presence of this nanoparticle catalyst. The work-up procedure of the reaction proceeded by “in-flask” extraction and allowed for easy recycling of the catalyst. By thermal gravimetric analysis the shelf life and catalyst stability could be measured. The reductant plays an important role for the bench stability of the nanoparticles, and hence methylmagnesium bromide was introduced to retain the catalytic activity.

#### Heterogeneous green protocols

**Scheme 4:**
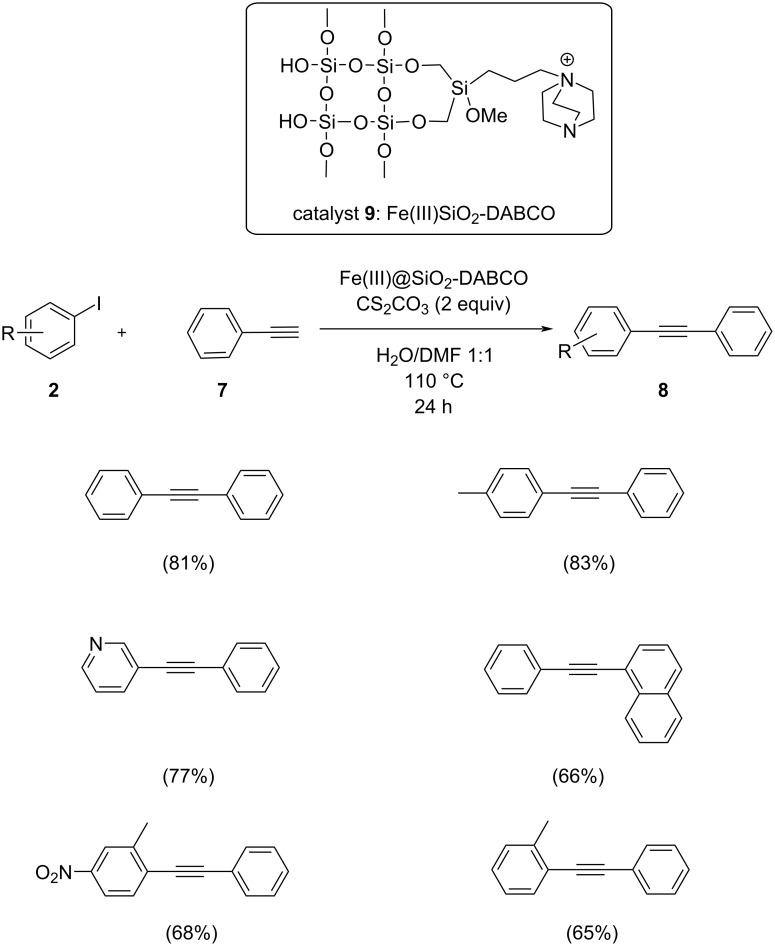
Sonogashira coupling catalyzed by a silica-supported heterogeneous Fe(III) catalyst.

**Scheme 5:**
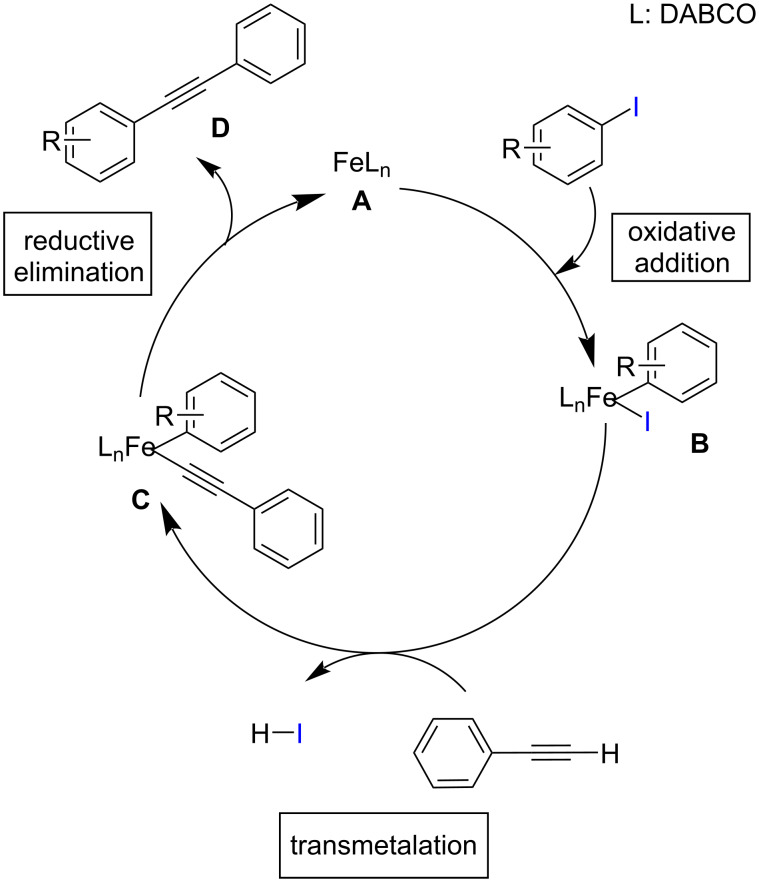
Suggested catalytic cycle for the Sonogashira coupling using a silica-supported heterogeneous Fe(III) catalyst.

Rizi and co-workers reported a silica-supported green heterogeneous Fe(III) catalyst for the cross-coupling reaction of aryl iodides and arylacetylenes in good yields (Scheme 4) [23]. The prepared Fe catalyst was found to be efficient and inexpensive, and could be recycled by filtration. This green strategy showed better yields in the presence of the inorganic base Cs_2_CO_3_ in DMF/H_2_O at 110 °C. Both electron-rich and electron-deficient aryl iodides showed good to excellent yields when coupled with phenylacetylene. The proposed mechanism is similar to the standard palladium-catalyzed Sonogashira reaction with the steps involving oxidative addition of the aryl/vinyl halide followed by transmetallation, and reductive elimination. The mechanism is shown in Scheme 5.

#### Non-green protocols

**Scheme 6:**
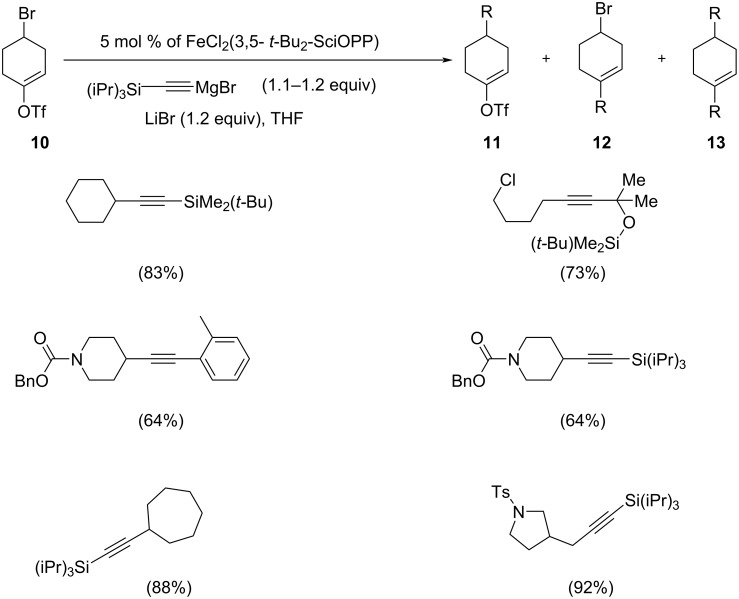
Chemoselective iron-catalyzed cross coupling of 4-bromo-1-cyclohexen-1-yltrifluromethane sulfonate with (*tert*-butyl dimethylsilyl)ethynylmagnesium bromide.

Nakamura et al. disclosed an Fe-catalyzed Sonogashira coupling between an alkynyl unit with primary and secondary alkyl halides (Scheme 6) [24]. They reported the coupling of an unactivated alkyl halide with the Fe catalyst can switch its chemoselectivity from C(sp^2^) to C(sp^3^). The reaction exhibited good yields, when a secondary alkyl bromide was treated with the alkynyl reagent. They performed the reaction between 4-bromo-1-cyclohexen-1-yltrifluromethane sulfonate and an alkynylmagnesium reagent in the presence of Fe catalyst with bisphosphine, which showed better yield of the coupling products. The optimized reaction conditions consisted of 5 mol % of the utilized catalyst with the dropwise addition of Grignard reagent, and 1.2 equivalents of LiBr as an effective additive in THF that resulted in good to excellent yields of the desired products.

**Scheme 7:**
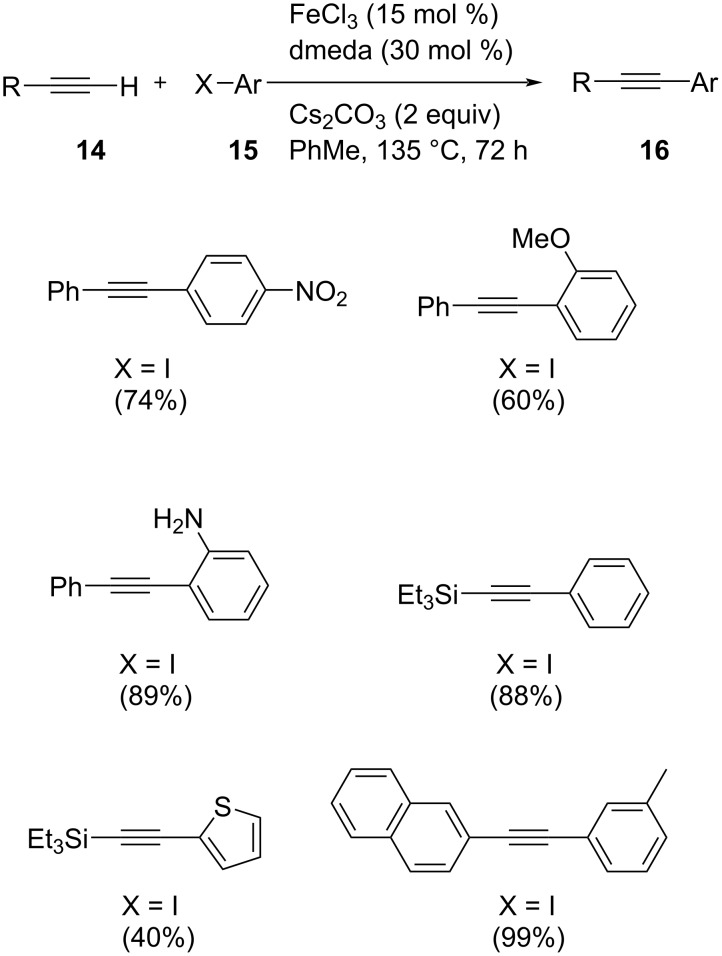
Fe-catalyzed Sonogashira coupling between terminal alkynes and aryl iodides.

**Scheme 8:**
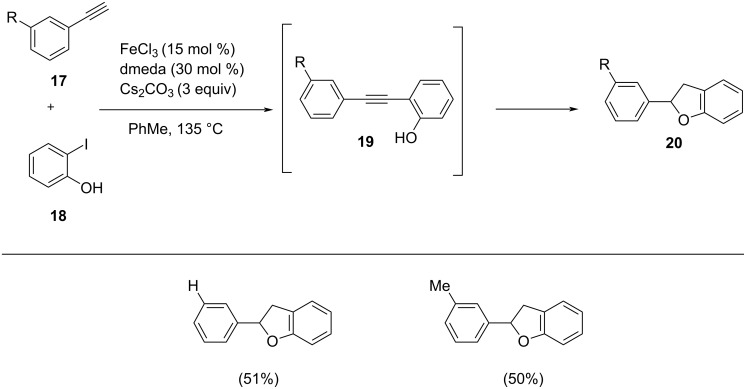
Iron-catalyzed domino Sonogashira coupling and hydroalkoxylation.

Bolm and co-workers reported a novel Fe-catalyzed cross-coupling for the arylation of terminal alkynes by using a combination of FeCl_3_ and *N*,*N*’-dimethylethylenediamine (dmeda) in catalytic amount [25]. A sample reaction in the absence of catalyst/ligand was also conducted, which yielded no desired product. Hence the role of iron catalyst in this transformation was found to be significant. The cross-coupling of different phenylacetylenes with phenyl iodide afforded various derivatives of diphenylacetylenes in excellent yields by using of 15 mol % of FeCl_3_ as catalyst, 2 equivalents of Cs_2_CO_3_ as base with 30 mol % of dmeda as ligand in toluene at 135 °C for 72 h (Scheme 7). On the other hand, acetylenes with alkyl substitution showed lower reactivity. In addition, to increase the importance of iron catalysts as a versatile tool in organic synthesis, a novel iron-catalyzed domino Sonogashira coupling/hydroalkoxylation of alkynes was also reported (Scheme 8).

**Scheme 9:**
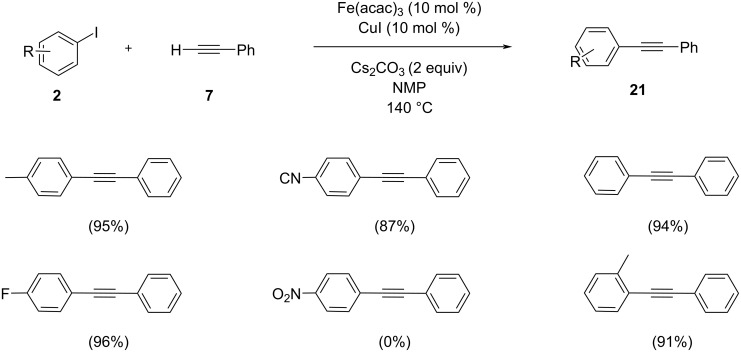
Sonogashira coupling of aryl halides and phenylacetylene in the presence of Fe(III) acetylacetonate as catalyst.

Vogel and co-workers demonstrated the Sonogashira cross-coupling reaction of aryl iodides with terminal alkynes by utilizing cheap, non-toxic iron salts and copper iodide (Scheme 9) [26]. The reaction of 4-iodotoluene with phenylacetylene was chosen as the model reaction without any external ligand and required 20 h for completion. However, the same reaction under microwave irradiation was complete within 2.5 h. The product was obtained with an excellent yield of 95% by the utilization of Fe(III) acetylacetonate and CuI as the catalyst. Among several iron salts tested Fe(III) acetylacetonate seemed to be the best catalyst. In order to outstretch the scope of the reaction phenylacetylene was coupled with various electrophilic partners.

**Scheme 10:**
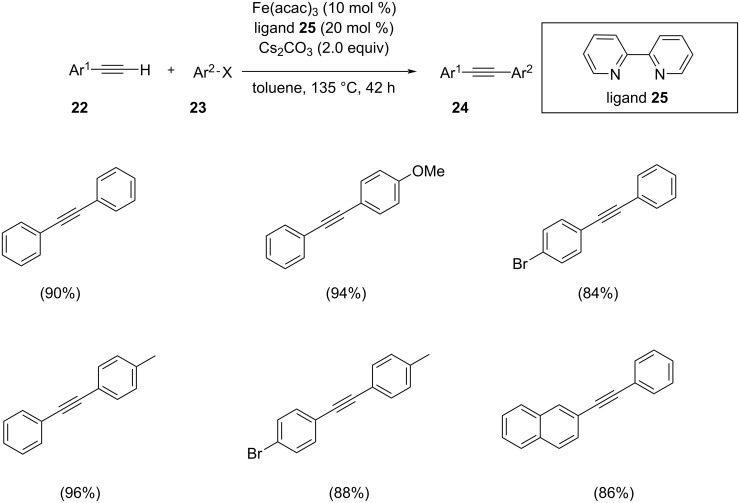
Sonogashira coupling of aryl iodides and alkynes with Fe(acac)_3_/2,2-bipyridine catalyst.

Liu et al. developed a catalytic system for the cross-coupling of aryl iodides with alkynes by the use of a combination of Fe(acac)_3_ and a ligand (Scheme 10) [27]. The optimized reaction of phenylacetylene with 1-iodobenzene comprised 10 mol % of Fe(acac)_3_, 20 mol % of 2,2’-bipyridyl and 2 equiv of Cs_2_CO_3_ in toluene at 135 °C for 42 h. By using Fe(acac)_3_ as catalyst and 2,2’-bipyridine (**25**) as the ligand the best product yield was obtained. The ligands tested in the cross-coupling reaction were shown to have a dramatic impact on the yield of the final product. Electron-rich aryl iodides showed higher yields than electron-deficient ones. In addition, 2-thiophenyl iodide also showed good yields of the products with various acetylenes. With regard to alkynes, 1-chloro-4-ethynylbenzene and 1-bromo-4-ethynylbenzene were found suitable substrates, which provided good to excellent yields of the products, and no homocoupling was observed in any reaction.

**Scheme 11:**
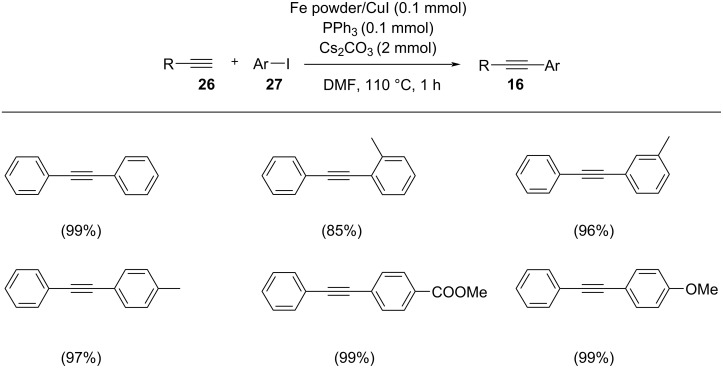
Sonogashira cross-coupling of terminal alkynes with aryl iodides in the presence of Fe powder/ PPh_3_/CuI catalyst.

Shim and co-workers demonstrated an efficient iron powder/CuI catalytic system for the cross-coupling of aryl iodides and vinyl, aryl or alkyl-substituted terminal alkynes [28]. They studied the reaction between phenylacetylene and iodobenzene in the presence of iron powder/CuI catalyst with PPh_3_ as ligand in DMF, which provided 95% yield of the desired product (Scheme 11). Lower yields were observed, when the reaction was carried out in the absence of either phosphine, CuI or Fe powder. Although the reaction proceeded in the absence of Fe powder, the yield of the alkyne product was lower than that by the addition of iron powder. Various substituted aryl iodides reacted with phenylacetylene to form the coupled alkyne products in excellent yield and the reaction displayed good functional group tolerance. The position of the substituents affected the yield of the product whereas the electronic nature had no significant effect. The yield of the coupled product was lower in case of an *ortho*-substituted aryl iodide than the yields obtained from *meta-* and *para-*substituted aryl iodides.

**Scheme 12:**
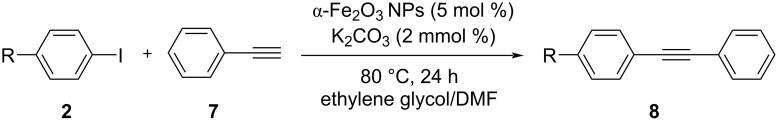
α-Fe_2_O_3_ nanoparticles-catalyzed coupling of phenylacetylene with aryl iodides.

**Scheme 13:**
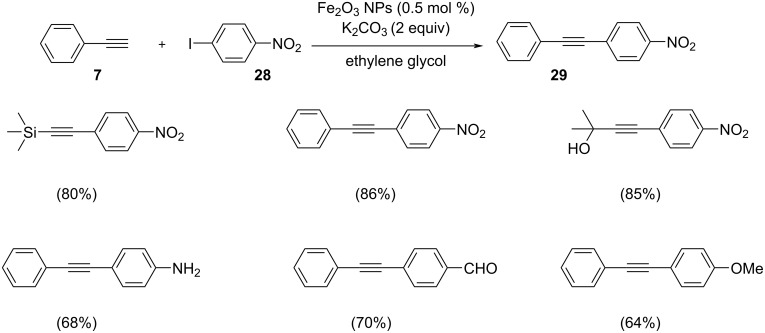
Sonogashira cross-coupling reaction between phenylacetylene and 4-substituted iodobenzenes catalyzed by in situ-generated α-Fe_2_O_3_ nanoparticles.

Kumar et al. reported a ferromagnetic α-Fe_2_O_3_-catalyzed coupling of phenylacetylene with aryl iodides (Scheme 12) [29]. They synthesized an azaindole-appended hexaphenylbenzene (HPB) moiety since it can easily undergo aggregation-induced emission enhancement (AIEE). The self-assembly of the HPB derivative resulted in the formation of fluorescent aggregates, which can act as a stabilizer or as a reactor for the development of α-Fe_2_O_3_ nanoparticles at room temperature. The catalytic efficiency of the α-Fe_2_O_3_ nanoparticles was found to be better than that of other metal catalysts usually used in Sonogashira coupling reactions. The nanoparticle catalyst can easily be separated by using magnet from the reaction mixture and is reusable. The proposed mechanistic step shows that the addition of Fe^3+^ ions into the solution directly interacts with the nitrogen atom of the hexaphenylbenzene (HPB)-based derivative and reduces the oxidation state from Fe(III) to Fe(0) which is further oxidized by the aqueous medium. Hence the aggregates of HPB can act as a good stabilizing agent. Similarly, Balla and co-workers discussed the scope of the reaction by the utilization of in-situ generated α-Fe_2_O_3_ nanoparticles with several activated and unactivated halides (Scheme 13) [30]. They also examined the effect of concentration of fluorescent aggregates of HPB, i.e., the size of the generated nanorods was found to increase with respect to Fe^3+^ ions-to-ligand ratio and vice versa. The reusability and separation of the ferromagnetic α-Fe_2_O_3_ nanoparticle catalyst were very effective, simple and economical due to its magnetic nature. The in situ-generated α-Fe_2_O_3_ nanoparticles showed better catalytic efficiency than other catalytic systems. For studying the efficiency of the catalyst, they selected the reaction of 4-iodonitrobenzene and phenylacetylene in the presence of 0.5 mol % of Fe_2_O_3_ nanoparticle as catalyst and K_2_CO_3_ as base in ethylene glycol at 80 °C. Both, the electrophilic character of the alkyl halide and C–H activation can be increased by the presence of active Lewis acid sites on the iron(III) nanoparticles. The scope for this catalytic system can be figured out by the presence of high temperature, high ligand concentration and activated ligands.

**Scheme 14:**
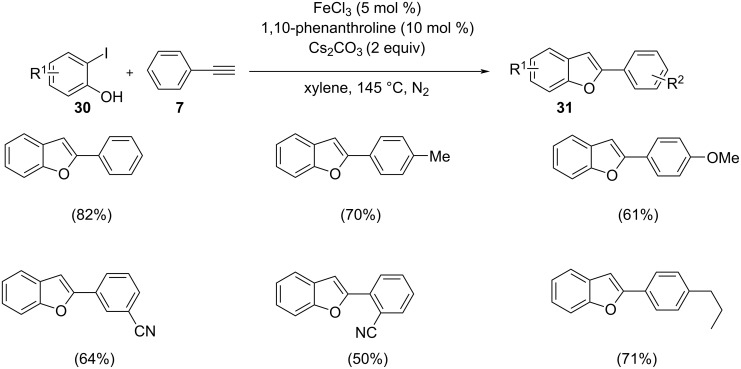
One-pot synthesis of 2-arylbenzo[*b*]furans via tandem Sonogashira coupling–cyclization protocol.

**Scheme 15:**
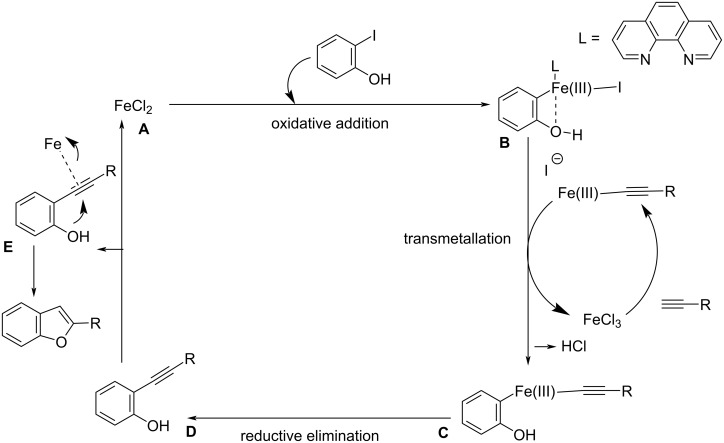
Suggested mechanism of the Fe(III) catalyzed coupling of *o*-iodophenol with acetylene derivatives.

Chen and co-workers synthesized 2-arylbenzo[*b*]furans by intramolecular arylation and Sonogashira cross-coupling of *o*-iodophenol with phenylacetylene/1-substituted-2-trimethylsilylacetylene under iron(III) catalysis in the presence of 5 mol % 1,10-phenanthroline as ligand and Cs_2_CO_3_ as base (Scheme 14) [31]. The use of 1,10-phenanthroline as ligand resulted in a shorter reaction time and better yield in comparison with the other ligands tested. Mechanistically, the iron is oxidized from Fe(II) to Fe(III) in the reaction step by the addition of 2-iodophenol which is further followed by transmetallation and reductive elimination. This synthetic protocol offers increased functional group tolerance and moderate to good yields of the products by the coupling of various functional group-substituted acetylenes with *o-*iodophenol. The proposed mechanistic study involves a Sonogashira coupling followed by a 5*-endo-dig* cyclization (Scheme 15). The precatalyst FeCl_2_ is activated under the reaction conditions which is denoted as complex **A**. The reaction begins with the oxidative addition of aryl/vinyl halide forming complex **B**. Iron acetylide on reaction with this complex in a transmetallation step yields complex **C**. The resulting product of reductive elimination regenerates the catalyst.

**Scheme 16:**
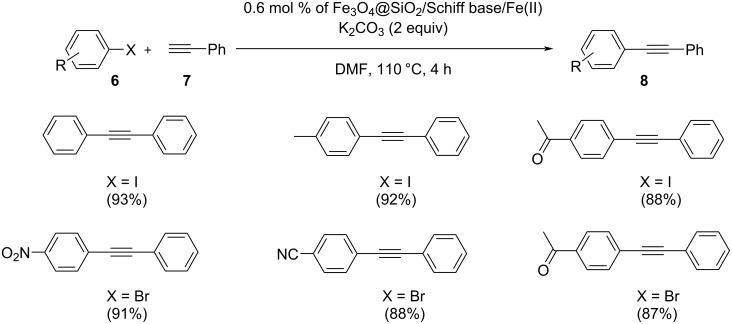
Fe_3_O_4_@SiO_2_/Schiff base/Fe(II)-catalyzed Sonogashira–Hagihara coupling reaction.

Javidi and co-workers reported a sequence of magnetically separable catalysts which consisted of Schiff base complexes of metal ions supported on superparamagnetic Fe_3_O_4_ nanoparticles (Scheme 16) [32]. To examine their catalytic activity, a Sonogashira coupling was carried out with these magnetic nanoparticles. The model substrates chosen were iodobenzenes and phenylacetylene that were reacted in the presence of 0.6 mol % of Fe_3_O_4_@SiO_2_/Schiff base complex of Fe(II) ions and 2 equivalents of K_2_CO_3_ in DMF at 110 °C for 4 h. The magnetically supported catalyst could be easily separated by an external magnetic field due to its paramagnetic nature, and it could be reused and recovered without loss of catalytic activity. The desired alkyne products were obtained in high yields. One of the most attractive features of this strategy was the finding that the morphology of the catalyst did not change after six consecutive reaction cycles.

**Scheme 17:**
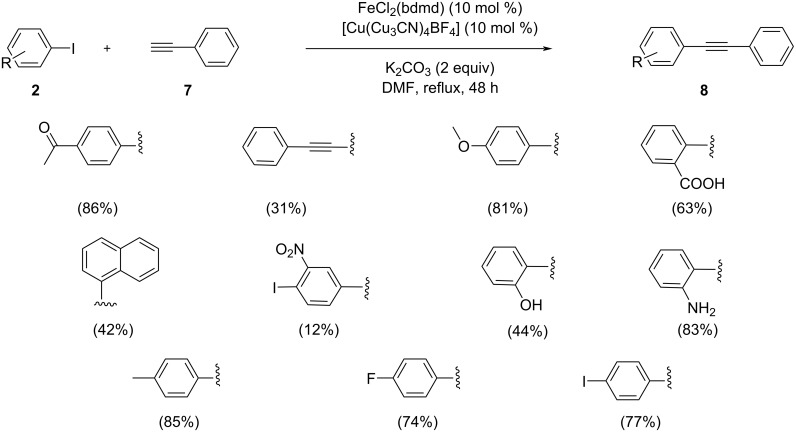
Sonogashira coupling using the Fe(II)(bdmd) catalyst in DMF/1,4-dioxane.

Apfel et al. reported the first selective cross-coupling reaction between terminal phenylacetylene and iodobenzene derivatives using Fe(II) bisphosphine complexes, which serve as precatalyst (Scheme 17) [33]. In the reaction *ortho*-iodo derivatives with an amino or alcohol functionality showed higher yields than the corresponding iodo derivatives with same substitutions in *meta* and *para* position due to the *ortho* directing effect. They then also analyzed the chance of applying the FeCl_2_(bdmd) catalyst for the Sonogashira reaction of iodobenzene derivatives having substituents which can potentially coordinate with iron and consequently cause catalyst deactivation. The efficacy of the coupling is also greatly reliant on the isomer utilized. FeCl_2_(bdmd) is very compatible with the reactive substrates having *para* substituents like iodo, fluoro, methyl, acetyl, and methoxy groups. The yield of the coupling product was observed to be decreased when the steric demand of aryl iodide species increased as in the case of the naphthyl-substituted iodo derivative.

**Scheme 18:**
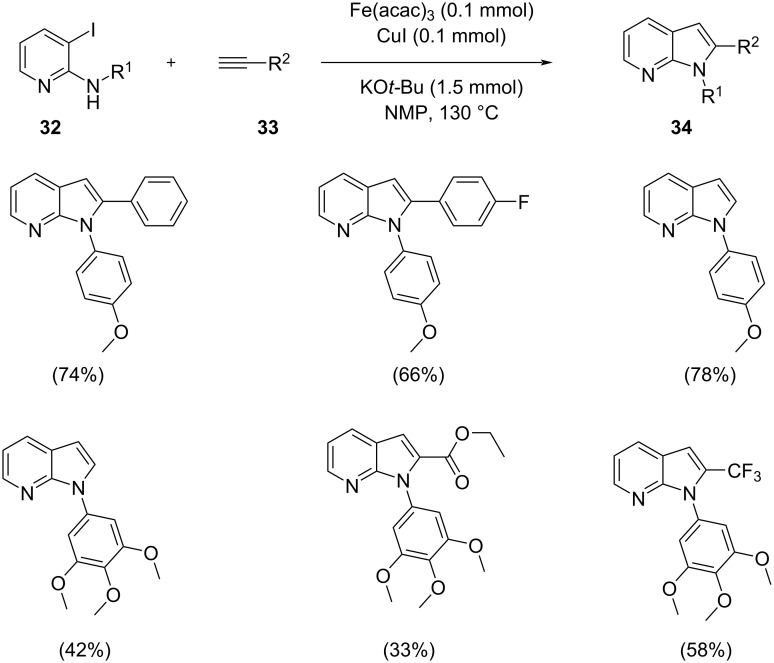
Synthesis of 7-azaindoles using Fe(acac)_3_ as catalyst.

**Scheme 19:**
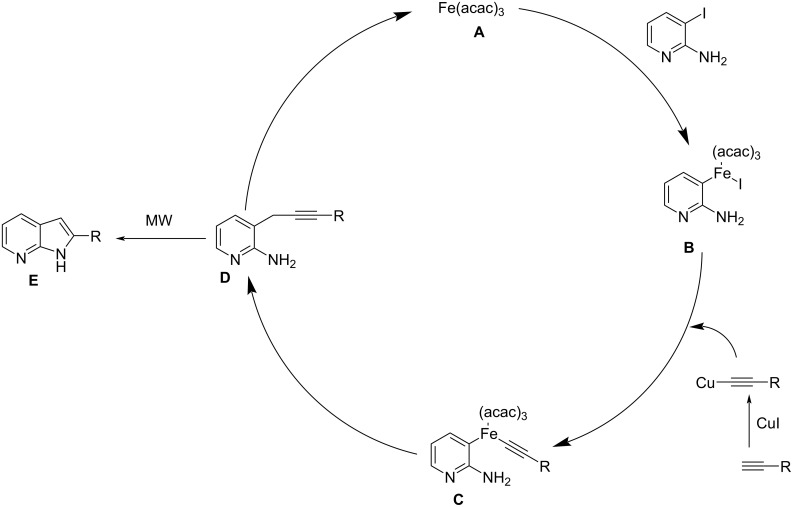
Plausible mechanistic pathway for the synthesis of 7-azaindoles.

An efficient method for the synthesis of 7-azaindole ring systems utilizing an iron catalyst under microwave irradiation has been reported [34]. *N*-Arylated azaindoles were prepared by Sonogashira coupling followed by cyclization. The Sonogashira coupling of 2-arylamino-3-iodopyridines with terminal acetylenes followed by cyclization afforded the 2-*N*-arylated 7-azaindoles. The optimized reaction conditions utilized 0.1 mmol of catalyst, 0.1 mmol of CuI and 1.5 mmol of KO*t*-Bu in NMP at 130 °C (Scheme 18). Substituents like -OCH_3_ on the arylacetylenes gave higher yields than acetylenes substituted with F, Cl, etc. The proposed mechanistic pathway shows that an organo iron complex **B** is formed by the oxidative addition of the iron catalyst to the pyridine derivative. Intermediate Fe species were obtained by transmetallation and finally a new carbon–carbon bond is formed by reductive elimination (Scheme 19). This method provided access to a diverse range of 7-azaindole derivatives under microwave irradiation that helps to reduce the reaction time and minimizes side product formation.

### Co-catalyzed Sonogashira cross-coupling reactions

#### Nanoparticle-based protocols

The advantages of using immobilized catalysts include the possibility of the catalyst to be easily recovered and reused, but it could also result in a reduced metal contamination of the products. However, most of the immobilized catalysts suffer from some problems like catalyst deactivation, low turnover numbers, and also leaching of metal.

**Scheme 20:**
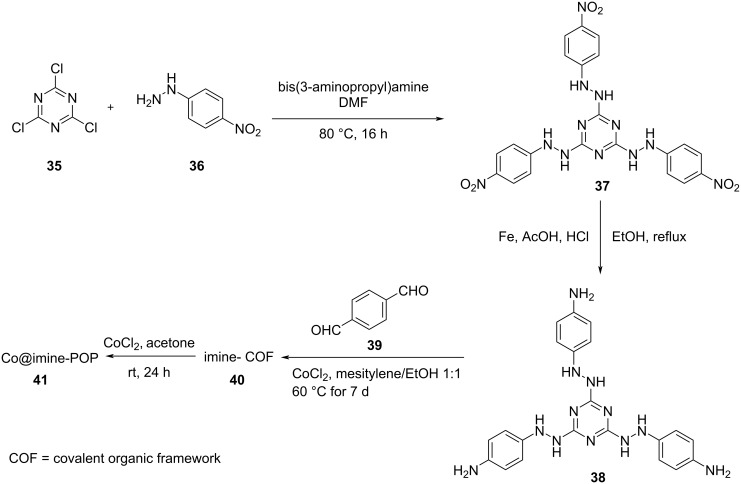
Synthesis of Co@imine-POP catalyst.

**Scheme 21:**
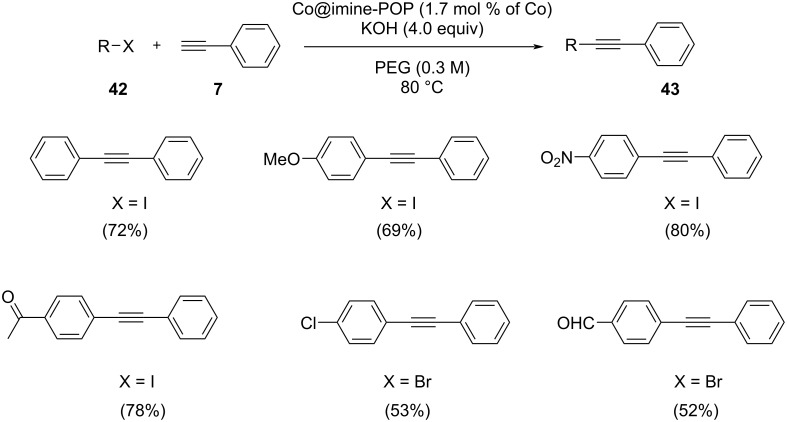
Sonogashira coupling of various arylhalides and phenylacetylene in the presence of Co@imine-POP catalyst.

A new heterogeneous cobalt catalyst (Co@imine-porous organic polymer) (Co@imine-POP) has been synthesized by immobilizing cobalt onto a nitrogen-rich porous organic polymer (Scheme 20) [35]. Using scanning electron microscopy, the surface morphology of the catalyst was evaluated. Nowadays, covalent organic frameworks (COF) were found to be a suitable support in catalysis. The catalytic activity of Co@imine-POP was investigated in a Sonogashira coupling of bromobenzene and phenylacetylene under different reaction conditions. The optimized reaction conditions were found to be 1.7 mol % of the Co@imine-POP catalyst, PEG as solvent and 80 °C reaction temperature (Scheme 21). The product yield was significantly lower only by decreasing the reaction temperature. The amino groups present on the POP backbone enable a suitable electronic surrounding of the cobalt species which promotes the reaction. The catalyst showed excellent recyclability for 8 successive runs without any remarkable decrease in its catalytic activity.

**Scheme 22:**
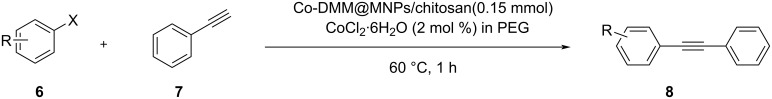
Sonogashira coupling of aryl halides and phenylacetylene using Co-DMM@MNPs/chitosan.

A novel cobalt catalyst with cobalt immobilized on nanochitosan fibers (Co-DMM@MNPs/chitosan) was fabricated using dimethyl malonate [36] and its applicability tested in a Sonogashira coupling reaction. The catalyst was found to be highly active, recyclable, and selective for the coupling between aryl halides and phenylacetylene in PEG as the solvent (Scheme 22). The effect of various reaction parameters such as solvent, base, temperature, and catalyst loading was assessed by the model reaction between bromobenzene and phenylacetylene. Optimization studies revealed PEG-200 and K_3_PO_4_ as suitable solvent and base, respectively. Aryl halides with electron-donating and electron-withdrawing groups afforded the corresponding products in good yields with 0.15 mmol of catalyst in PEG at 60 °C.

**Scheme 23:**
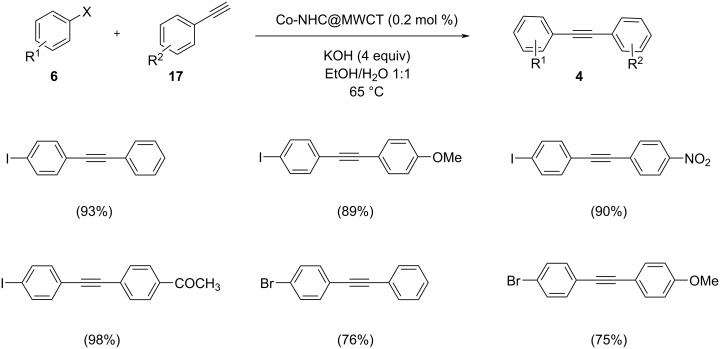
Sonogashira cross-coupling of aryl halides with terminal acetylenes in the presence of Co-NHC@MWCNTs.

**Scheme 24:**
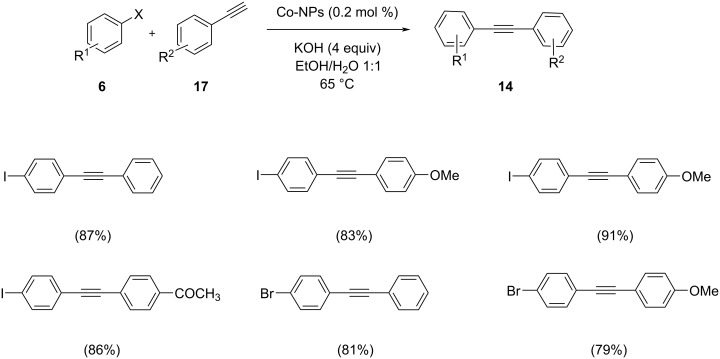
Sonogashira cross-coupling of aryl halides with terminal acetylenes in the presence of Co nanoparticles.

A three-component coupling of alkyne with a heterogeneous complex of cobalt and N-heterocyclic ligand supported on carbon nanotubes and pure cobalt nanoparticles was developed [37]. Propargylamine derivatives were synthesized in extremely high yields in green solvents by this catalyst. The catalytic system can be easily recovered and reused 7 times without any decrease in its activity. For comparison, the Sonogashira reaction of phenylacetylene and iodobenzene was performed in the presence of the two catalytic systems Co-NHC@MWCNTs and Co-NPS (Scheme 23 and Scheme 24). The reaction proceeded with four equivalents of KOH in EtOH/H_2_O at 65 °C. Aryl iodides showed higher reactivity and less active aryl chlorides were successfully coupled to give the desired products in acceptable yields. The reaction carried out by both catalysts gave excellent yields of up to 98% for Co-NHC@MWCNTs and a still good yield of up to 91% for reactions catalyzed by Co-NPs. It was clearly shown that the complex of Co-N-heterocyclic ligand exhibits a higher activity.

**Scheme 25:**
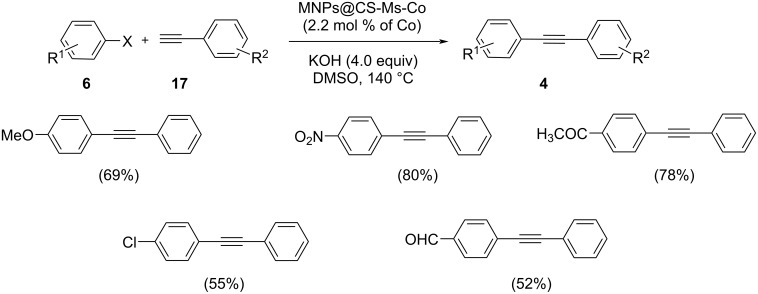
Sonogashira coupling reaction of aryl halides with phenylacetylene in the presence of Co nanoparticles.

Sonogashira cross-coupling reactions were also reported by making use of a heterogeneous, efficient and green cobalt catalyst obtained through complexation of cobalt with methyl salicylate-functionalized chitosan fibres (Scheme 25) [38]. The coupling products by reaction of phenylacetylene and various aryl halides were obtained in moderate to good yields in the presence of this green catalyst with 4 equivalents of KOH as the base in DMSO at 140 °C. Without any decrease in the catalytic activity, the catalyst could be recovered by applying an external magnet and reused for successive five runs. Functional groups including nitro, carbonyl, and methoxy on the aryl halide were compatible with the catalyst. Aryl iodides gave better yields when compared to aryl bromides. A good synergistic effect of the nanoparticles with the ligand achieved the high activities of the Co-MS@MNPs/CS.

**Scheme 26:**
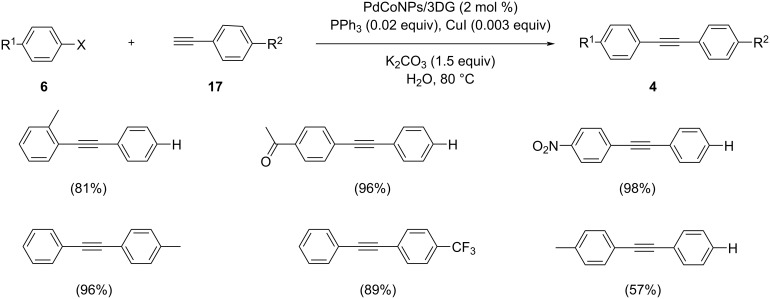
PdCoNPs-3DG nanocomposite-catalyzed Sonogashira cross coupling of aryl halide and terminal alkynes.

Taking aryl halides and terminal alkynes as the reactants, Vajargahy and Dabiri discussed a facile method for a Sonogashira coupling reaction catalyzed by Pd/Co bimetallic nanoparticles supported on three-dimensional graphene (PdCoNPs-3DG) in aqueous medium under aerobic conditions (Scheme 26) [39]. The graphene support was prepared by oxidation of graphite, treatment of the resulting graphene oxide with ethylene diamine in an autoclave followed by freeze-drying to obtain three dimensional graphene with a high nitrogen content. Heating of the latter with salts of Pd(II) and Co(II) in ethylene glycol and water at pH 11 afforded the desired PdCo nanoparticles-3D graphene nanocomposite. The catalyst was then studied in the Sonogashira coupling reaction of arylacetylenes and aryl halides in the presence of various bases and solvents. The maximum yield was obtained when PdCoNPs/3DG was used with PPh_3_ as ligand and K_2_CO_3_ as base in water at 80 °C for 8 h. The reaction exhibited a high selectivity and the catalyst could be reused for at least seven cycles.

**Scheme 27:**
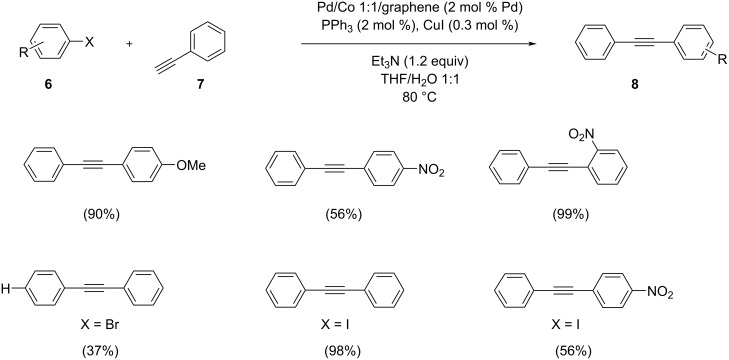
Sonogashira cross-coupling of aryl halides and phenylacetylene in the presence of graphene-supported Pd/Co nanoparticles.

Xu et al. analyzed a facile method for the coupling reaction of aryl halide and phenylacetylene in the presence of a series of PdCo bimetallic nanoparticles with different compositions on graphite sheets as highly active catalysts (Scheme 27) [40]. The catalyst was prepared by a chemical reduction method and characterized by various techniques like Raman, XRD, TEM, and XPS. The Pd/Co(1:1)NPs were found to be the most active catalyst for the reaction in a THF/water mixture with triethylamine as base at 80 °C. The catalyst exhibited a high selectivity, which may be related to the presence of the cobalt species. The increase in reactivity was assigned to both the large surface area of graphene and the promotional effect of Co-dopants which lay out more Pd active site for the reactants.

**Scheme 28:**
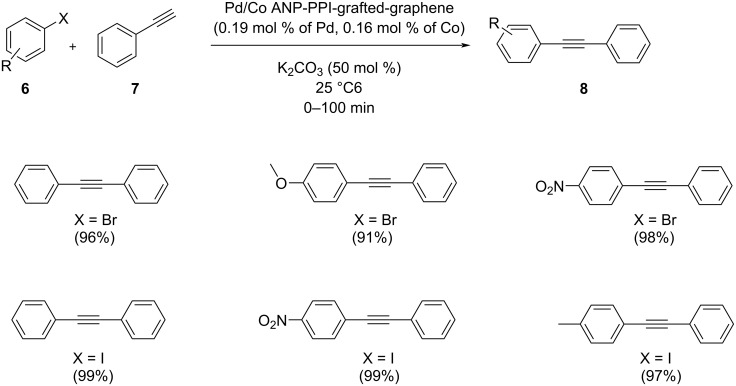
Sonogashira cross-coupling with Pd/Co ANP-PPI-graphene.

Mahyari et al. reported a facile, efficient, and environmentally friendly protocol for the coupling of aryl halides and phenylacetylene using a diverse strategy. Third generation polypropyleneimine dendrimers were grown on graphene nanosheets for catalyzing the reaction using highly active Pd-Co bimetallic nanoparticles under copper and solvent-free conditions at room temperature. K_2_CO_3_ was found to be the suitable base which provided a relatively good yield (Scheme 28) [41]. The graphite sheets could control the size of nanoparticles and the catalyst was stable at room temperature. Higher stability and an easy recovery of the catalyst are the advantages of using graphene as the support. The model reaction was done between phenylacetylene and iodobenzene to evaluate the catalytic properties of the graphene-supported catalyst and resulted in a good yield, ease of reaction work-up, and compliance with green chemistry principles.

**Scheme 29:**
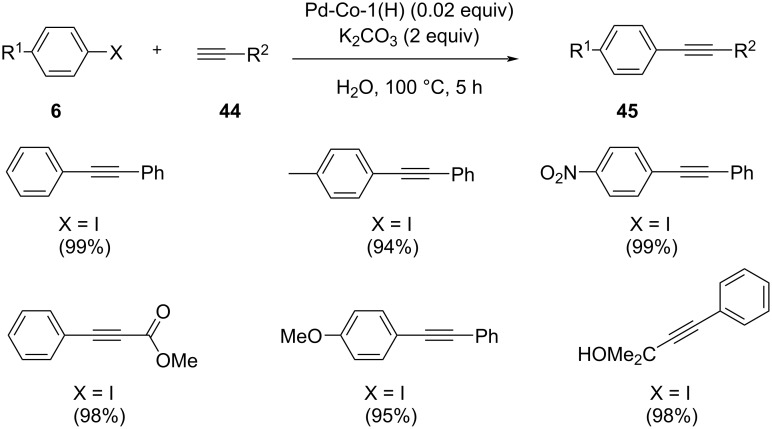
Pd-Co-1(H)-catalyzed Sonogashira coupling reaction.

A Sonogashira-type coupling reaction between aryl halides and terminal alkynes in aqueous medium in the presence of hollow palladium-cobalt bimetallic nanoparticles was reported by Li et al. (Scheme 29) [42]. The reaction conditions involved a catalyst amount containing 0.02 mmol of Pd and CuI as co-catalyst at 100 °C. The catalytic activity of the Pd-Co nanospheres could be controlled by changing their composition (Pd-Co-x(H) hollow, with x indicating the molar ratio of Co^2+^ and Pd^2+^ in the solution). The difference in reactivity was shown by reacting aryl halides and terminal alkynes containing electron-withdrawing or electron-donating groups. The catalyst could be reused after seven successive runs which displays the nature of Pd active sites that did not change after being repetitively used. Further, the catalyst can be easily separated from the reaction mixture through a centrifugation method. With an increasing Co content, the iodobenzene conversion to diphenylacetylene over Pd-Co-x(H) first increased and then decreased. The promotional effect of cobalt dopants on the activity of the catalyst was assigned on the basis of their dispersing effect on active sites of Pd. The maximum conversion was acquired on Pd-Co-1(H).

**Scheme 30:**
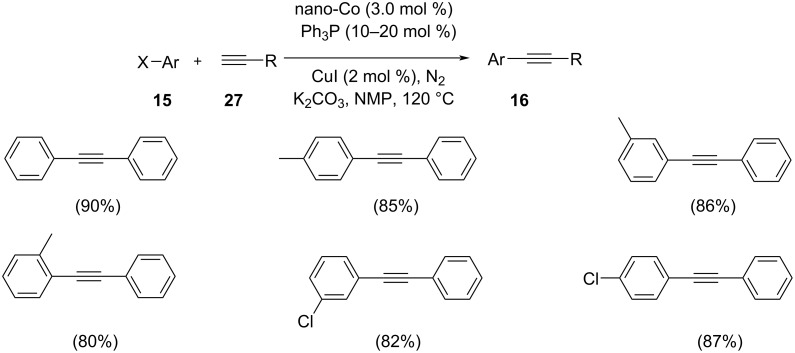
The coupling of aryl halides with terminal alkynes using cobalt hollow nanospheres as catalyst.

**Scheme 31:**
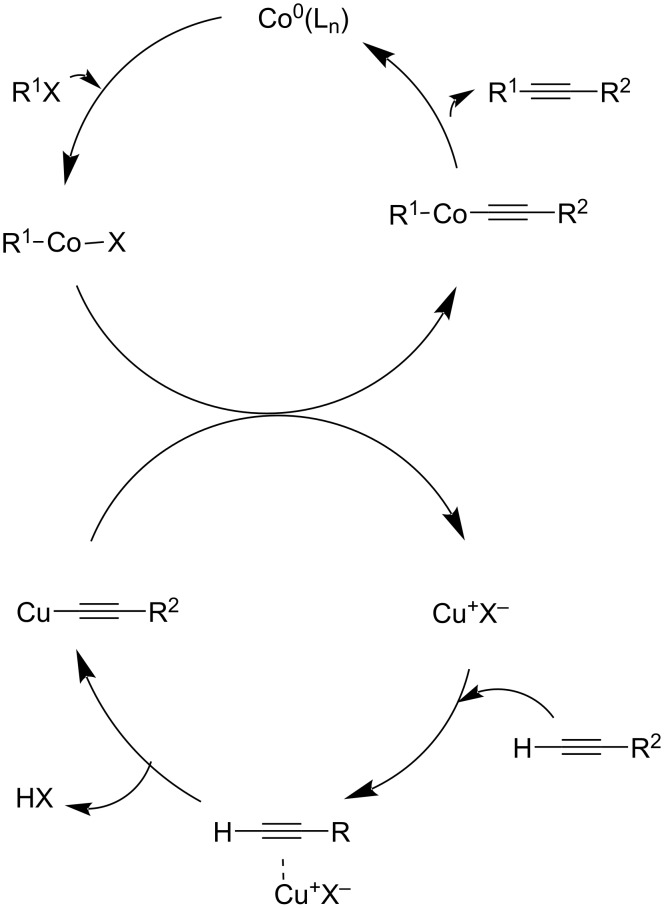
A plausible mechanism for the cobalt-catalyzed Sonogashira coupling reaction.

A Sonogashira reaction of aryl iodides with terminal alkynes catalyzed by cobalt hollow nanospheres has been developed by Bao and co-workers (Scheme 30) [43]. The reaction was carried out in the presence of nitrogen atmosphere at 120 °C using NMP as the solvent. The experiment suggested that 3 mol % of the catalyst and 2 mol % of copper co-catalyst could catalyze the reaction sufficiently. The large surface area of the hollow spheres results in high catalytic activity. The reaction was insensitive to the electronic characteristics of the substituents present on the aryl halide. The reaction of aliphatic alkynes and aryl halides with lower boiling point proceeded at low temperature thus avoiding volatilization of the substrate. The catalyst could be reused three times after being separated from the reaction mixture and was washed thoroughly with diethyl ether and methanol. To avoid the oxidative coupling of terminal alkynes the reaction was carried out in presence of nitrogen atmosphere. The authors also proposed a mechanism for the reaction (Scheme 31).

#### Non-nanoparticle-based protocols

Nanoparticles are materials with sizes around nanometer scales. The used nanoparticles are environmentally friendly magnetic cross-linked chitosan fibers which is a natural polysaccharide polymer. In this regard the use of such polymers is promising due to a reduced toxicity and low cost. In addition, multiwalled carbon nanotubes are also used as desirable polymers. However, nanoscale materials may pose a considerable health risk due to the gradual increase in surface area per mass unit.

**Scheme 32:**
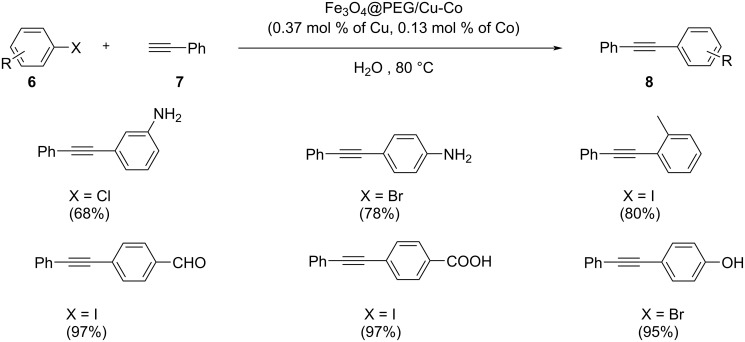
Sonogashira cross-coupling reaction of arylhalides with phenylacetylene catalyzed by Fe_3_O_4_@PEG/Cu-Co.

**Scheme 33:**
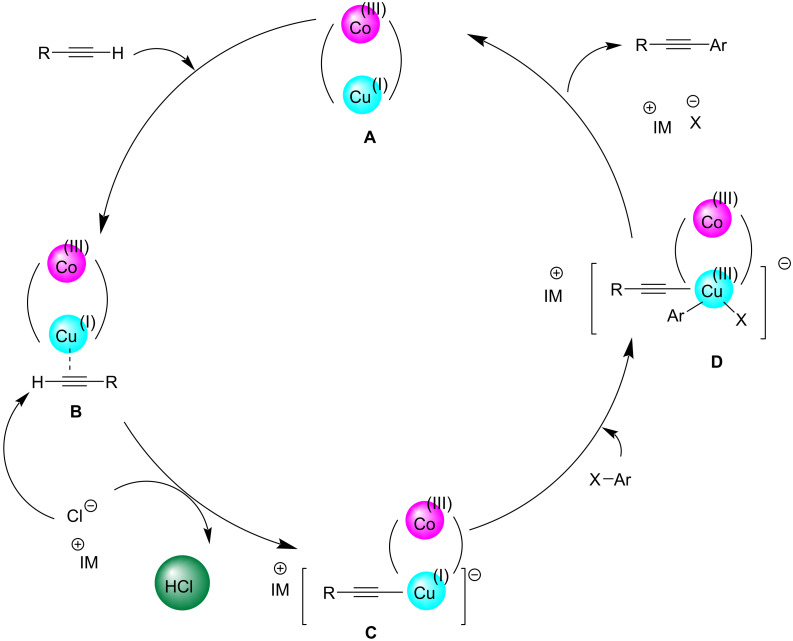
Plausible mechanism of Sonogashira cross-coupling reaction catalyzed by Fe_3_O_4_@PEG/Cu-Co.

A novel type of environmentally benign, economical, and recyclable bimetallic magnetic particles called Fe_3_O_4_@PEG/Cu-Co, was found to be effective in catalyzing the Sonogashira coupling of aryl halides and phenylacetylene in water as the solvent (Scheme 32) [44]. Aryl halides containing functional groups such as amino, hydroxy, carboxy or formyl, were found compatible with the catalyst and afforded the products with high yield. The efficiency of the catalyst in water was found to be increased by introducing polyethylene glycol (PEG) chains. From the mechanistic explanation, the reaction proceeds via an oxidative addition and reductive elimination route. Fe_3_O_4_@PEG/Cu-Co catalyst transfers an electron from Co(III) to Cu(I). As the next step, a π complex **B** is formed between the metal center and the acetylene groups. A copper acetylide intermediate **C** is formed from complex **B**. The hydrophilicity of the catalyst could be increased by the use of polyethylene glycol groups. In addition, the aryl halide oxidatively adds to copper changing its oxidation state to +3. Finally, the desired product is formed by reductive elimination and the catalyst returns to the cycle (Scheme 33). The designed catalyst was characterized by different techniques which showed various features like structure, morphology, magnetic nature, and catalytic performance. One among the major attractions of this protocol is that the catalyst could be reused for at least 7 successive runs.

**Scheme 34:**
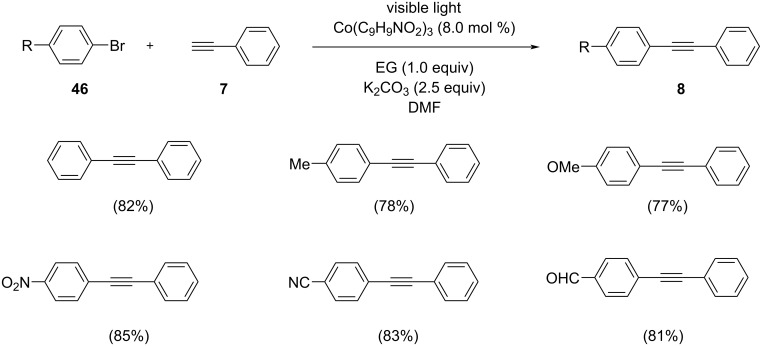
Sonogashira coupling reaction of *para*-substituted bromobenzenes with phenylacetylene in the presence of Co(C_9_H_9_NO_2_)_3_.

Chu et al. developed an efficient and inexpensive procedure for the formation of carbon–carbon bonds. This protocol involves the visible light-assisted Sonogashira coupling of aryl bromides and terminal alkynes in the presence of the Co(C_9_H_9_NO_2_)_3_ complex as the catalyst (Scheme 34) [45]. This novel catalytic system provided 23 alkyne products in a substrate scope study using ethylene glycol as additive and K_2_CO_3_ as base in DMF under visible light irradiation. The reaction of Co(OAc)_2_·4H_2_O with 2-(hydroxyimino)-1-phenylpropan-1-one in EtOH resulted in the formation of the Co catalyst. The coupling strategy exhibited only poor activity by the use of pure Co salts and the reaction was promoted by the addition of 2-(hydroxyimino)-1-phenylpropan-1-one. This reaction displayed promising yields by the use of ethylene glycol as additive. Aryl bromides with electron-donating and electron-withdrawing substituents in the *para* position gave the desired coupled products in moderate to excellent yields. With *p-*bromobenzylcyanide and 1-bromonaphthalene as substrates, the reaction afforded medium yields of the products after 10 h. In addition, 3,5-ditrifluromethylbromobenzene and 3,4-dibenzyloxybromobenzene were used as the substrates and coupled with 4-ethylphenylacetylene affording the products with 69% and 66% yield, respectively.

**Scheme 35:**
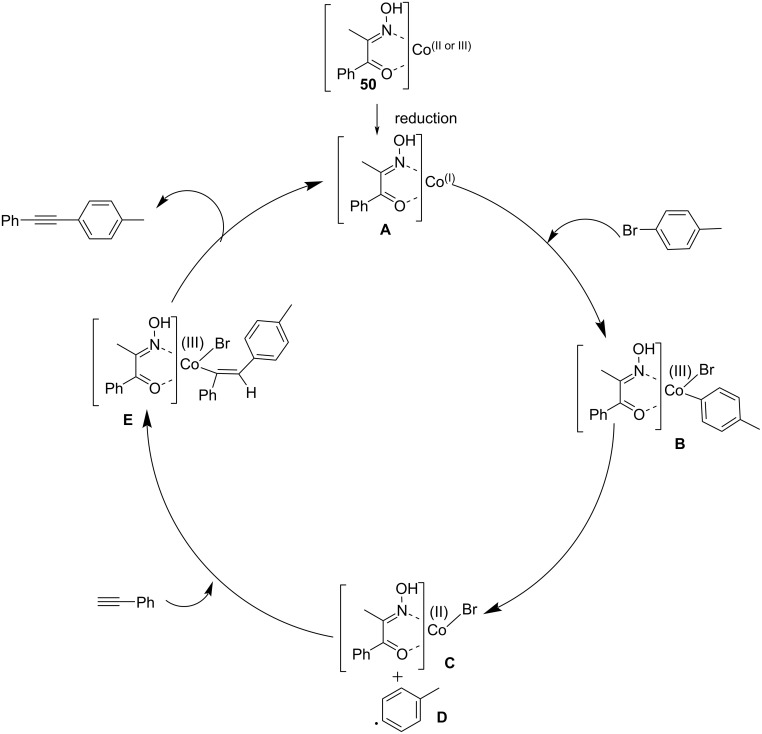
Possible mechanism for the visible light-assisted cobalt complex-catalyzed Sonogashira coupling. (Reproduced with permission from *ChemCatChem*. **2018**, *10*, 758–762.)

The mechanistic scenario begins with the reduction of cobalt(II/III) complex **B** to form cobalt(I) complex **A** in the Sonogashira reaction system. The cobaloxime derivative Co(C_9_H_9_NO_2_)_3_ complex is used as a radical precursor. The reaction of preformed cobalt(I) complex **A** with an electrophile results in the formation of a cobalt(III) intermediate. Subsequent homolytic cleavage and generation of cobalt(II) complex **C** and radical **D** the radical undergoes addition to phenylacetylene to provide cobalt(III) intermediate **E**. The targeted compound 1-methyl-4-(2-phenylethynyl)benzene is obtained by reductive elimination in the presence of ethylene glycol and regeneration of the cobalt(I)N complex to recommence the catalytic cycle (Scheme 35).

**Scheme 36:**
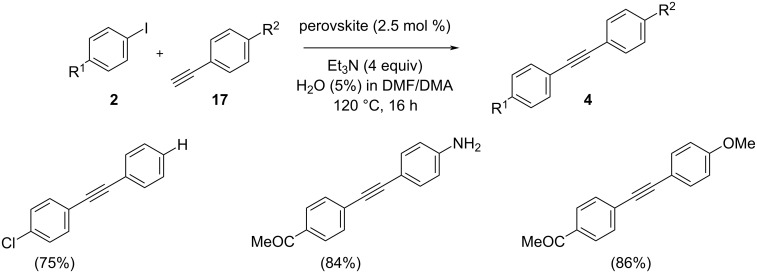
Sonogashira cross-coupling of aryl halides and phenylacetylene using cobalt as additive.

**Scheme 37:**
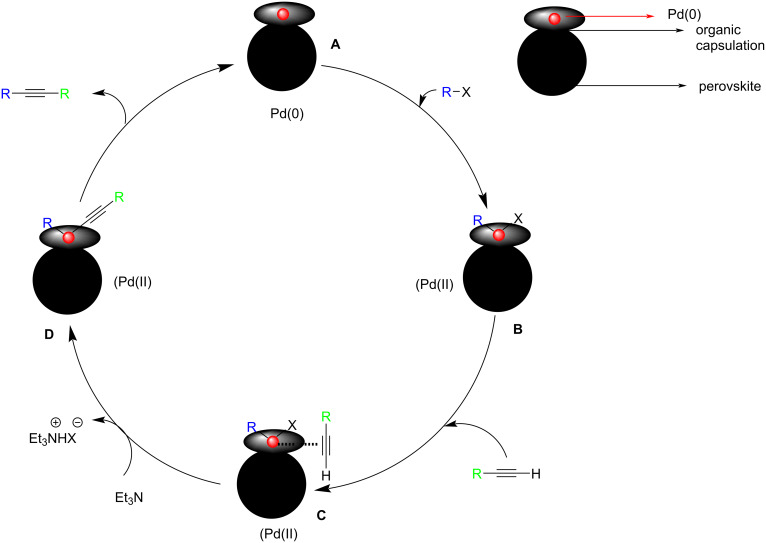
Plausible mechanism of Sonogashira cross-coupling reaction over [LaPd*]. (Reproduced with permission from *ACS Sustainable Chem. Eng.* **2019**, *7*, 12697–12706.)

Ley and co-workers disclosed the utility of a cobalt-containing perovskite, LaFe_0.57_Co_0.38_Pd_0.05_O_3_ [LaPd^*^] as a good catalyst (Scheme 36) [46]. The catalyst was found to be efficient to promote the Sonogashira coupling of arylhalides and phenylacetylene under microwave and conventional heating conditions. Rather than applying a set of reaction conditions, the desired reaction could be achieved by the modification of the catalyst. Various reactant pairs were treated with 2.5 mol % of one out of 3 palladium-containing perovskites, with Et_3_N in a solvent mixture of 5% H_2_O in DMF/DMA at 120 °C. Furthermore, the presence of hydrophobicity on the surface of the LaCoO_3_ catalyst can improve the interaction between the precursors of the Sonogashira coupling reaction and the catalyst. A mechanism was also suggested where this in situ formed colloidal layer acts as the anchoring agent (Scheme 37).

## Conclusion

The Sonogashira reaction is an important coupling reaction in organic chemistry. Even though most of the reactions are well explored by the use of expensive palladium metal, innumerable procedures have been elaborated to restore expensive, rare and often toxic noble metal catalysts by the much more abundant green metals. This review summarizes the use of cheap, abundant, non-toxic transition metals such as iron and cobalt in the Sonogashira coupling because of their natural abundance and environmentally friendly behavior. The iron-catalyzed reaction has been further classified on the basis of the nature of catalyst, and it can be considered as a green footstep since it uses green catalytic systems. The use of such green variants can reduce the negative impacts on both the environment and health factors. As a developing field of organic synthesis, greener areas have to be explored further and to the best of our knowledge, this review is the first attempt in this area.
